# Recruiting individuals from disadvantaged neighborhoods into a local recruitment registry

**DOI:** 10.1002/alz70859_105817

**Published:** 2025-12-25

**Authors:** Megan G Witbracht, Thuy V Lu, Dan Hoang, Amy J.H. Kind, Daniel L Gillen, Josh D Grill

**Affiliations:** ^1^ Institute for Memory Impairments and Neurological Disorders, University of California, Irvine, Irvine, CA USA; ^2^ University of California, Irvine, Irvine, CA USA; ^3^ The UC Irvine Institute for Memory Impairments and Neurological Disorders, Irvine, CA USA; ^4^ Center for Health Disparities Research, University of Wisconsin School of Medicine and Public Health, Madison, WI USA; ^5^ Department of Medicine, Geriatrics Division, University of Wisconsin School of Medicine and Public Health, Madison, WI USA

## Abstract

**Background:**

Numerous populations are underrepresented in Alzheimer’s disease (AD) research. Recruitment registries are a tool to accelerate research accrual that may also hold promise to increase diversity and inclusion in AD research. The Area Deprivation Index (ADI) is a robust indicator of neighborhood health that has been linked to brain health outcomes. This study examined strategies for recruiting individuals from diverse neighborhoods, based on ADI, into a local recruitment registry, the UC Irvine Consent‐to‐Contact (UCI C2C).

**Method:**

We conducted an interrupted time series experiment to test the effectiveness of social media advertising and mailed postcards across quintiles of ADI in Orange County CA. Every 8 weeks for 12 periods, beginning in February 2023, we launched recruitment interventions, balanced by ADI, promoting the purpose and attributes of the UCI C2C Registry. Postcards 5.75” by 10.75” in size were mailed to pre‐specified Zip+4 regions. Meta advertisements with similar imagery and messaging but optimized for Facebook and Instagram targeting prespecified zip codes were published for 7 days. We used chi‐squared tests for independence to determine if enrollment differed among the ADI quintiles for each intervention.

**Result:**

Among 88,289 individuals sent postcards by mail and 901,776 users reached through Meta advertisements,113 (0.1%) people and 74 (0.01%) people, enrolled in the registry, respectively. We observed no difference in enrollment by ADI among those enrolling through the postcard intervention (*X* ^2^ (0,113) = 5.540, p = 0.236); whereas those enrolling through Meta demonstrated differential rates by ADI quintiles (*X* ^2^(0, 74) = 38.297, *p*‐value < 0.001) with people living in neighborhoods of the lowest deprivation enrolling at higher frequency.

**Conclusion:**

Relative to social media advertisements, mailed postcards enrolled a higher proportion of individuals and were more effective at recruiting people equally across levels of neighborhood disadvantage into a local recruitment registry.

